# A Review of the Potential Mechanisms of Action of Baclofen in Alcohol Use Disorder

**DOI:** 10.3389/fpsyt.2018.00506

**Published:** 2018-10-17

**Authors:** Renaud de Beaurepaire

**Affiliations:** Groupe Hospitalier Paul-Guiraud, Villejuif, France

**Keywords:** GABA receptor b, reward network, amygdala, Pavlovian associations, substitution (morphine, methadone, buprenorphin)

## Abstract

Baclofen, a GABA-B receptor agonist, is a promising treatment for alcohol use disorder (AUD). Its mechanism of action in this condition is unknown. GABA-B receptors interact with many biological systems potentially involved in AUD, including transduction pathways and neurotransmitter systems. Preclinical studies have shown that GABA-B receptors are involved in memory storage and retrieval, reward, motivation, mood and anxiety; neuroimaging studies in humans show that baclofen produces region-specific alterations in cerebral activity; GABA-B receptor activation may have neuroprotective effects; baclofen also has anti-inflammatory properties that may be of interest in the context of addiction. However, none of these biological effects fully explain the mechanism of action of baclofen in AUD. Data from clinical studies have provided a certain number of elements which may be useful for the comprehension of its mechanism of action: baclofen typically induces a state of indifference toward alcohol; the effective dose of baclofen in AUD is extremely variable from one patient to another; higher treatment doses correlate with the severity of the addiction; many of the side effects of baclofen resemble those of alcohol, raising the possibility that baclofen acts as a substitution drug; usually, however, there is no tolerance to the effects of baclofen during long-term AUD treatment. In the present article, the biological effects of baclofen are reviewed in the light of its clinical effects in AUD, assuming that, in many instances, clinical effects can be reliable indicators of underlying biological processes. In conclusion, it is proposed that baclofen may suppress the Pavlovian association between cues and rewards through an action in a critical part of the dopaminergic network (the amygdala), thereby normalizing the functional connectivity in the reward network. It is also proposed that this action of baclofen is made possible by the fact that baclofen and alcohol act on similar brain systems in certain regions of the brain.

## Introduction

Baclofen is a gamma-aminobutyric acid (GABA) analog that activates the GABA-B receptor subtype, and is used worldwide in neurology for the treatment of spasticity due to its myorelaxant properties (1). Many preclinical [see (2), for review] and clinical studies have demonstrated the efficacy of baclofen in the treatment of several addictive disorders, including alcohol use disorder (AUD) (3–10), even though negative results have also been published (11, 12). While it is clearly established that the myorelaxant properties of baclofen are related to a dampening of the spinal motor reflex (13), its potential mechanism of action in AUD remains elusive. Central GABA-B receptors are involved in the regulation of a large number of systems and functions, including several neurotransmitter systems (dopamine, serotonin, norepinephrine, glutamate), transduction pathways, memory, and other cognitive functions, as well as inflammation. All these systems and functions are possibly involved in the anti-addictive effects of baclofen. In the present paper, the biological effects of baclofen are reviewed in the light of its clinical effects, assuming that, in many instances, clinical effects can be reliable indicators of underlying biological processes.

The effects of baclofen in the treatment of alcohol dependence have been thoroughly described by a physician suffering from AUD, Olivier Ameisen, who reported the cure of his alcohol dependence with a high dose of baclofen, first in an article published in 2005, then later in a book published in 2008 (3, 14). He reported that, in his case, the dose of 270 mg/day produced a state of “complete indifference” toward alcohol. Indifference is not an operational concept in addictology. It is nevertheless a concept that should be taken into consideration. It is characterized by an effortless suppression of craving, but goes beyond it. In people indifferent to alcohol, the experience of drinking or seeing alcohol cues has changed completely, as if alcohol had no meaning to them anymore. Those who are indifferent to alcohol can drink a glass of an alcoholic beverage, they do not finish the glass, they do not want to continue drinking, they feel nothing, while they remain unchanged for other aspects of their life, which they enjoy normally. The state of indifference is not transitory when the effective dose of baclofen is maintained: on the contrary, it is very long lasting, and people completely indifferent to alcohol can generally stop baclofen after one or a few years of treatment, and they most often do not relapse (as if all memories associated with alcohol had vanished). This differs from those who have been cured by using other methods, for whom abstinence most generally requires a lot of effort, and for whom craving for alcohol often returns when they resume some alcohol drinking or see alcohol cues. However, all AUD patients treated with baclofen do not reach such a state of complete indifference. My experience (more than a thousand patients treated with a high-dose of baclofen over the last 10 years) is that about one third of the patients reach this state of complete indifference; while another third experiences a clear decrease in craving, but not its complete suppression (these patients drink very significantly less, but still have moments of desire for alcohol); in the last third of patients baclofen treatment is ineffective despite often reaching very high doses. In these latter patients, the dose increase may have been limited by adverse effects, but it happens that some patients reach very high doses (superior to 400 mg/day) without achieving a state of indifference. In any event, a state of indifference can be reached in a substantial number of patients, and one of the aims of the present article is to try to address the concept of indifference in biological terms.

AUD is a chronic relapsing disorder characterized by an increased motivation to seek alcohol and drink compulsively, with an increasing loss of control over drinking, progressing from impulsivity to compulsivity (15). In clinical research, the concept of compulsion is generally not used: the word compulsion is absent from the DSM-5 AUD section, where the term *craving*, defined as “a strong desire or urge to use alcohol” (16), seems to encompass the compulsion to drink. According to the DSM-5, AUD is commonly associated with anxiety, depression, psychotic disorders, cognitive disorders and sleep disorders. Impulsivity is cited as a vulnerability factor for AUD. Regarding biological research, addiction models use the concept of craving (associated with those of preoccupation/anticipation), and add the concepts of positive and negative reinforcement to those of impulsivity and compulsivity. In positive reinforcement, cues and contexts associated with drinking acquire incentive salience after repeated association with drinking, and increasing strength of salience progressively leads to compulsive alcohol seeking and drinking. In negative reinforcement, craving is induced by the motivational value of the negative states of alcohol withdrawal. This leads to a state of preoccupation/anticipation where craving is intensified by the anticipation of access to alcohol resulting in compulsive alcohol seeking and drinking. A challenge in alcohol research is to explain how specific cues or contexts are paired with states of craving, while explaining which mechanisms of brain encoding are involved in these associations. It is hypothesized that the understanding of how specific neuronal ensembles encode and mediate the recall of learned associations among the cues, contexts, and behaviors during alcohol seeking and drinking will be helpful for the comprehension of how baclofen works in the treatment of AUD (17).

As mentioned above, baclofen is a selective GABA-B receptor agonist. GABA-B receptors are heterodimeric metabotropic receptors consisting of one GABA-B receptor-1 subunit (GABBR1) and one GABA-B receptor-2 subunit (GABBR2). GABA-B receptors are coupled via G-proteins to potassium and calcium channels, and to adenylate-cyclase (18). Studies have shown that GABA-B receptors are highly expressed all throughout the brain, some regions having a very high density of receptors, other regions a low or a very low density, and some regions insignificant densities. The variable densities of GABA-B receptors may have important implications regarding the use of baclofen in the treatment of AUD, given that, when a patient takes baclofen activation of regions with high densities should have clearer and more immediate physiological and behavioral consequences than the activation of regions with low densities. Binding and expression of GABA-B receptors have been studied in rodents. The Chu et al. binding study (using baclofen) showed highest densities of GABA-B receptors in the medial habenula, thalamus, cerebellum, cortex and colliculus; while ventral tegmental area and mesolimbic dopaminergic projections were among the structures with the lowest binding (19). Bowery et al. reported highest densities of GABA-B binding in the cerebellum, interpeduncular nucleus, frontal cortex, and thalamus (20), and highest GABBR1 mRNA transcripts in the hippocampus, thalamus, and cerebellum (21). Billington et al. (22), using immunohistochemistry targeting GABBR1 and GABBR2, found receptor colocalization in the cerebellum, hippocampus, cortex, thalamus, and basal ganglia; the raphe nucleus was strongly stained for GABBR1 and weakly for GABBR2; there was no significant staining in the VTA and mesolimbic dopamine projections. Lu et al. reported strong expression of GABBR1 in the medial habenula, hippocampus, hypothalamus (supraoptic and suprachiasmatic nuclei), and cerebellum; intermediate expression in thalamus and brainstem nuclei containing monoaminergic neurons; and low expression in globus pallidus, ventral pallidum, and substantia nigra pars reticulata (23). Li et al. (24) reported strong expression of both GABBR1 and GABBR2 in medial habenula, cingulate and piriform cortex, cerebellum, and hippocampus; moderate expression in thalamic nuclei, amygdala, and other parts of the cortex; low expression in the basal ganglia and hypothalamus; insignificant expression in the ventral mesencephalon, where the VTA is located (24). Conversely, a study using immunohistochemistry and focusing selectively on midbrain monoaminergic nuclei showed that GABA-B receptors are present in neurons of the VTA and raphe nuclei (no comparison in terms of density of receptors was made with other parts of the brain) (25). Besides, acute treatment with baclofen in rats produces an activation of a number of brain nuclei, mostly in the hypothalamus, the amygdala, and the brainstem, and has no detectable effect in reward-relevant regions such as the nucleus accumbens, striatum, or ventral tegmental area (26). In conclusion, despite some discrepancies between these studies and despite the fact that it is not known to what extent observations made in rodents are relevant to humans, it is likely that, when given to patients, baclofen action is much stronger in brain structures that contain high or very high densities of GABA-B receptors, such as the cerebellum, medial habenula, hippocampus, some nuclei of the hypothalamus and thalamus, and certain parts of the cortex, than in dopaminergic structures.

Adverse effects generally occur before anticraving effects during baclofen treatment of AUD, especially when baclofen is used in order to make patients reach a state of complete indifference, for which high or very high doses are most often necessary. Many adverse effects may in large part be explained by an early occurring activation of brain areas containing high densities of GABA-B receptors. For example, the most common and early occurring baclofen adverse effects include fatigue, diurnal somnolence and nocturnal insomnia. Theses symptoms may be explained by an action of baclofen on GABA-B receptors in the brainstem and hypothalamus, which are among the structures most strongly activated by baclofen, and which control basic states of vigilance (in particular through the suprachiasmatic nucleus). Frequently occurring adverse sensory effects (tinnitus, paresthaesias, blurred vision, etc.) may be explained by the high density of receptors in the thalamus, and memory problems by the high density of receptors in the hippocampus. Baclofen can also frequently promote anxiolysis; this could possibly be explained by an early occurring effect of baclofen on serotonin neurons and on the amygdala. Hyperactivity of serotonin raphe neurons or hyperactivity of the amygdala are mechanisms known to produce anxiety; baclofen acutely inhibits serotonin neurons and serotonin release (27, 28) while short-term baclofen treatment inhibits amygdala reactivity to incentive cues (29). Most importantly, it has recently been shown that alcohol addiction is associated with impaired GABA clearance in the amygdala, with an increase in GABA tone associated with higher anxiety-like behavior (30). Baclofen could possibly improve anxiety through a rapid normalizing effect on amygdala GABA tone. Clinical studies have shown that baclofen has anxiolytic effects in patients with AUD (31) and it has been hypothesized that the anticraving effects of baclofen could be related to an anxiolytic effect (5, 6, 32). A study by Morley et al. showed no anticraving effect in AUD patients, but a secondary analysis showed that baclofen was effective in the group of anxious patients included in the study (33). Given that craving for alcohol is closely related to states of stress (34, 35), the stress-relieving effects of baclofen may contribute to reduce craving in AUD patients. In addition, activation of GABA-B receptors could normalize the abnormal GABA tone in the amygdala and have significant therapeutic effects (36). However, adverse and anxiolytic effects generally occur during the first days or weeks of treatment, while a state of complete indifference most often occurs much later, after one or several months of treatment (depending on the dose needed and the protocol of dose increase). This could imply that complete indifference to alcohol does not result from an immediate or short-term effect of baclofen on GABA-B receptors, but is rather the result of long-term plastic remodeling of certain brain systems.

Preclinical studies have highlighted such plastic effects of baclofen or other GABA-B agonists on brain systems after chronic treatment. Keegan et al. have shown that rats treated chronically with baclofen have significant decreases in G-protein-dependent signal transduction (measured by GTP-gamma-S binding) in the frontal cortex, septum, amygdala, and parabrachial nucleus (37). Such decreases demonstrate a general desensitization of G-protein-dependent systems. But chronically treated rats also show signaling alterations via kinase cascades, including increases in activation of focal adhesion kinase (FAK) and of activated glycogen synthase kinase 3 (GSK3ß), and elevations in phosphorylated dopamine- and cAMP-regulated phosphoprotein-32 (DARPP-32), in several brain structures, which could indicate an absence of desensitization in these structures (i.e., no tolerance). According to Keegan et al. neuroadaptation mediated by G-proteins correlates with tolerance, while signaling alterations via kinase cascades shows cross-talk between GABA-B receptors and alternative mechanisms that are resistant to desensitization. Regarding brain areas, chronic baclofen treatment produced a sustained increase in auto-phosphorylation of FAK in the caudate, an increase in phosphorylations of GSK3ß in the caudate and putamen, and an increase of DARPP-32 in the nucleus accumbens. These actions demonstrate that chronic baclofen can induce significant plastic effects in dopaminergic structures, and that the effects of baclofen in these dopaminergic structures are associated with an absence of tolerance. The Keegan et al. study shows that chronic baclofen treatment also produces a sustained increase in kinase cascades activity in other regions, namely the cortex, thalamus, and hippocampus for FAK; the cortex, thalamus and septum for GSK3ß; and the cortex, thalamus, hippocampus and amygdala for DARPP-32. These regions are not primarily dopaminergic, but nevertheless receive dopaminergic projections, and, for many of them, they are part of the reward network (38). In summary, chronic baclofen produces G-protein desensitization in a certain number of structures, and alterations in signaling via kinase cascades, with resistance to desensitization, in a set of structures that are closely related to the dopamine network. It may be hypothesized that structures showing desensitization could be involved in the adverse effects of baclofen, which all tend to vanish over time, while structures showing no desensitization are involved in the therapeutic effect on baclofen in AUD—these last structures being possibly involved in the indifference toward alcohol. Indeed, when baclofen produces a complete indifference toward alcohol, there is no tolerance to this effect [no requirement for “markedly increased dose […] to achieve the desired effect,” as tolerance is defined in the DSM-5 (16)]; on the contrary baclofen can be progressively decreased over time in patients indifferent to alcohol, until, for many patients, a possible discontinuation of the treatment after a more or less lengthy period of time.

## The anti-dopaminergic hypothesis

All addictive substances alter dopaminergic signaling in the mesocorticolimbic system, and models of addiction posit positive and negative reinforcement as closely linked to dopaminergic reward systems (39). In positive reinforcement, an increase in drinking is associated with increases in the release of dopamine in the brain, producing a feeling of pleasure. Whereas in negative reinforcement, alcohol is taken to alleviate a negative emotional state, and is associated with decreased dopamine in the striatum. And it is widely accepted that, in patients with chronic AUD, reward thresholds are increased and dopamine function is decreased, leading to a general “dopamine-impoverished” brain (40). The enduring reduction of dopaminergic systems activity in patients' brains logically implies that drugs used in the treatment of AUD should enhance dopamine function to restore brain circuits disrupted by alcohol use. The idea of a strict hypo-dopaminergic state in the brain of AUD patients remains controversial (41). However, dopamine antagonists have never shown effectiveness in the treatment of AUD (42). Furthermore, preclinical studies have shown that baclofen inhibits dopamine transmission (43–48). Such dopamine antagonist properties do not *a priori* posit baclofen as a good candidate for the treatment of subjects who have a dopamine-impoverished brain. However, some studies have shown that baclofen could have dose-dependent effects on dopamine systems, with low doses increasing dopamine transmission and high doses inhibiting it. This has been demonstrated in animals (49), in *in vitro* cell preparations (50), and in humans (51).

Studies investigating the effect of baclofen in animal models of addiction show that systemic administration of baclofen reduces the acquisition and maintenance of alcohol consumption (52–55), motivation to drink (56), binge-like drinking (57), relapse-like drinking (58), severity of alcohol withdrawal signs (53), cue-induced reinstatement of previously extinguished alcohol-seeking behavior (59), and the reinforcing and motivational properties of alcohol (60–65) in different validated rodent models of AUD [for review, see (2)]. That baclofen reduces alcohol consumption in animal models has been further strengthened by the demonstration that R(+)-baclofen, and not S(-)-baclofen selectively reduces self-administration of alcohol in rats (66). Regarding the mechanism of action of baclofen in these models, it is generally hypothesized that baclofen reduces alcohol consumption through an anti-dopaminergic effect. The hypothesis is based on two major points. The first is the fact that baclofen has clear anti-rewarding effects. These effects have been shown not only for alcohol consumption, but also for the consumption of cocaine (67), amphetamine (68), and even of non-drug reinforcers such as sucrose, saccharin, or regular food pellets, suggesting that baclofen produces a generalized suppression of reward-motivated behaviors (2). The second is that microinjection of baclofen directly into the VTA blocks the behavioral response to cues and the cue-evoked firing of subpopulations of NAc neurons that respond to predictive cues (69). Baclofen and other GABA-B agonists microinjected into the VTA suppress alcohol-seeking behavior (70), alcohol consumption (71), and alcohol-induced conditioned place preference (72). Microinjection of baclofen in the nucleus accumbens also decreases binge-like alcohol drinking (73). Given that the microinjection of baclofen in the VTA dampens the activity of dopaminergic neurons, it has been hypothesized that baclofen may exert its anti-addictive properties by means of its ability to reduce the activity of dopaminergic neurons (74).

From a clinical standpoint, this hypothesis is not entirely satisfying because the clinical effects of baclofen in AUD patients are not really those of an antidopaminergic effect. Indeed, acute administration of baclofen may produce sedation, and sedation could be related to an inhibition of dopaminergic systems, but baclofen often produces a behavioral disinhibition, and quite frequently an evident hypomania (approximately 15% of patients). Disinhibition could be possibly linked to a dose-related effect of baclofen on dopaminergic neurons, where a low dose of baclofen activates VTA neurons and a high dose inhibits them (50, 51). However, the experience of baclofen treatment in patients with AUD shows that most often the stimulant effect is not dose-dependent; it appears at any dose, and sometimes at high doses. And when a disinhibitory effect occurs at a low dose, it is almost never followed by a state of inhibition when doses are increased, as should be the case if baclofen had a dose-dependent biphasic effect. In baclofen-treated patients, a disinhibitory effect is generally accompanied by a sensation of well-being, or euphoria, or even by a hypomanic state; and it is well established that these feelings or symptoms are associated with an increase in striatal dopamine activity (75–77). On the other hand, inhibition of dopaminergic systems has been consistently related to apathy, anhedonia and depression (78). Baclofen's ability of to produce major depression during treatment of AUD is a subject of discussion [see (79)]; if this were the case, it is certainly very rare. However, baclofen makes patients sometimes feel dull, apathetic, and joyless (approximately 15% of patients), suggesting, rather, a state of atypical depression that does not meet the criteria of a major depressive episode. This state is possibly related to a hypo-dopaminergic state. Thus, baclofen can produce states of disinhibition/hypomania or of apathy, in about 30% of patients. The large majority of patients never experience these symptoms. This shows that baclofen can have an effect on dopaminergic systems during treatment of AUD patients, but this effect can be that of a hyper- or a hypo-dopaminergic effect, and that it occurs in a minority of patients. More importantly, the clinical experience shows that the states of sedation, apathy, disinhibition, or hypomania have no relation to the therapeutic effect of baclofen; they occur independently of an anti-craving effect, and these symptoms are commonly considered as side-effects of baclofen. As a whole, these elements are not compatible with a general hypothesis that would posit an inhibition of dopaminergic systems as a central mechanism for the anti-addictive action of baclofen (Table 1).

**Table 1:** Correspondence between potential mechanisms, symptoms and neurobiological substrates.

**Mechanism**	**Symptom**	**Neurobiological substrate**	**Method**	**References**
Dopamine	Anticraving effect Sedation/apathy Hypomania/mania Indifference	↘ dopamine in VTA-NAc ↘ dopamine in VTA-NAc ↗ dopamine in striatum Long-term remodeling of dopamine circuits?	Intra-VTA B inject Peripheral B inject Brain imaging	(2) (review) (44) (75)
Connectivity	Inhibits reinstatement Eth seeking decrease Eth relapse Anticraving effect Anticraving effect AUD treatment Indifference	Inactivation of infralimbic cortex in rats (similar to anterior cingulate in humans) Inactivation of baso-lateral amygdala Inactivation of ventral subiculum Stimulation of dorsal anterior cingulate Stimulation of the nucleus accumbens Multiple networks Gaba tone in the amygdala?	Gaba inhibition Gaba inhibition Gaba inhibition TMS DBS Naltrexone Baclofen	(80) (81) (82) (83) (84) (review) (85)
Substitution	Similar effects of Eth and GABA-B activation General symptoms Anxiolysis Withdrawal GHB deficiency hypothesis	Multiple brain structures Brain GHB receptors	Eth and GABA-B agonists studies GHB studies	(86); (87) (16); (88) (89); (90) (16); (91) (92)

*B, baclofen; Eth, ethanol; VTA-NAc, Ventral Tegmental Area-Nucleus Accumbens; Gaba inhibition, local injection of baclofen+muscimol; TMS, Transcranial Magnetic Stimulation; DBS, Deep Brain Stimulation. References, only the first author is cited; Two references on the substitution line, the first refers to alcohol, the second to baclofen or other GABA-B agonists*.

Another reason why the therapeutic effect of baclofen in AUD is likely not to be related to a global anti-dopaminergic effect is that it is not compatible with the phenomenology of the state of indifference toward alcohol. Patients indifferent to alcohol are no longer interested in alcohol, but they experience normal enjoyment for the other aspects of their life. People who enjoy life normally necessarily have intact reward systems. The state of indifference is always reached after treatment has lasted for a certain amount of time, which is likely a period of remodeling brain circuits. It cannot be excluded that that an initial blockage of the dopaminergic systems participates in the remodeling of brain circuits. The above-mentioned study by Keegan et al. shows that chronic baclofen treatment induces plastic changes in a number of structures and systems, most of which are part of, or are closely linked with, the brain's reward network.

## Long-term network alterations

The concept of indifference is not a scientific concept, while those of craving and compulsive drinking are such concepts. Craving/compulsive consumption of alcohol occurs after repeated consumption of alcohol, likely in relation with complex adaptations in brain circuits. The challenge is to explain how a chronic treatment with a GABA-B agonist is able to overcome these numerous and complex brain adaptations, and to lead to a state of indifference toward alcohol. The different components of the progressive set-up of the compulsive drinking behavior involve neural substrates that belong to the dopaminergic reward network. Schematically, the reward network has three major components (38): a VTA-ventral striatum/nucleus accumbens system (VTA-NAc) that encodes stimuli valence; a VTA-amygdala/hippocampus system (which in the amygdala includes the basolateral and central nuclei) that forms associative related memories; And a VTA-medial prefrontal cortex system (VTA-mPFC) that regulates executive control. Studies have shown that craving occurring in response to alcohol cues is associated with the activation of structures, which, for the most part, belong the reward system. These structures are the nucleus accumbens/ventral striatum; the anterior, posterior and dorsal cingulate cortex; the orbitofrontal cortex; the dorso-medial prefrontal cortex; the amygdala, the hippocampus and para-hippocampus; and the cerebellum (93–95). These different structures are interconnected through complex networks. Preclinical models have demonstrated that a direct inactivation of some of these structures can suppress craving or the reinstatement of drinking. For instance, it has been shown that inactivation of the prelimbic cortex inhibits ethanol self-administration (80); that the inactivation of the baso-lateral amygdala attenuates context-induced alcohol-seeking (81); or that the inactivation of the ventral subiculum decreases context-induced relapse to alcohol seeking (82). Therefore, inactivation of localized parts of the reward network may globally inhibit craving or compulsive drinking. Studies in AUD patients have shown the same kind of results. Transcranial Magnetic Stimulation (TMS) targeted to the dorsal anterior cingulate cortex (dACC) has been shown to reliably suppress craving (83, 96); a similar effect has been found with Deep Brain Stimulation (DBS) targeted in the nucleus accumbens (84). It is well established that TMS and DBS efficacy are related to their ability to change network connectivity (97).

Many studies have shown that AUD is associated with abnormal brain connectivity. It seems, in a simplified way, that in AUD patients connectivity is increased in regions that are involved in appetitive drive and reduced in regions that mediate executive control, while in long-term abstinent patients activity is decreased in reward circuitry and increased in executive control regions (98–102). This is however a simplified view of the question, which is certainly far more complex (103–108). But the important point in the context of the present article is to highlight that there are dysfunctional networks in the brains of AUD patients, and to try to understand how effective pharmacological treatments used in AUD can normalize these dysfunctional networks. The literature dealing with the effects of pharmacological treatments for AUD on brain connectivity is scarce. A study by Morris et al. (85) showed that AUD patients have heightened local efficiency of neural networks, indicating disturbances of information processing—more isolation and clustering of functionally related regions, stronger processing in certain regions, with less cross-talk between distinct functional processes—, and that naltrexone, a commonly used treatment of AUD, can normalize these abnormalities. Gamma-hydroxybutyrate (GHB), a medication used in the treatment of AUD that activates GABA-B receptors, has been shown to markedly alter functional connectivity in healthy volunteers (109). Generally speaking, it is very likely that all effective AUD treatments, whether pharmacological, psychological, or using local stimulation, do so by normalizing functional connectivity, possibly leading to a decrease in the strength of appetitive networks and to an increase of that of executive control regions, with a recovery of balanced cross-talk between the different local functions.

It is therefore hypothesized that chronic baclofen treatment produces a state of indifference through a normalization of brain network connectivity. Chronic baclofen produces many changes in the brain that could impact connectivity. We have previously mentioned that chronic baclofen produces plastic changes in regions of the reward system, including desensitization in G-protein-dependent systems and alterations in signaling of several kinase cascades (FAK, GSK3ß, DARPP-32) that are resistant to desensitization (37). In addition, AUD is associated with marked brain neuro-immune alterations (110); and studies have shown that baclofen has anti-inflammatory and neuroprotective effects on the brain. Baclofen attenuates neuro-inflammation (111) and inflammatory signaling (112); inhibits the release of pro-inflammatory cytokines from microglia (113) and from astrocytes (114); and decreases oxidative stress (111); interestingly, baclofen is an allosteric modulator of CXCR4, a receptor for the chemokine CXCL12, which has been causally involved in several neurological disorders, including stroke, brain tumors, HIV encephalopathy and multiple sclerosis (115). GABA-B-receptor activation alters also the activity of dopamine, serotonin, norepinephrine, GABA and glutamate, which are prominent neurotransmitters implicated in alcohol dependence and are involved in the modulation of brain networks. It is not known whether these effects of baclofen on neurotransmitter or neuroimmune factors can alter functional connectivity in AUD, but it has been shown that neuroimmune/neurotransmitter dysregulation in other psychiatric disorders, such as bipolar disorder, disrupt local brain network connectivity and have deleterious effects on the brain, and that these effects can be treated with appropriate pharmacological treatments (116). Abnormal glutamate release and function have been found in the brains of AUD patients and glutamate and/or GABA neurotransmission may underlie resting-state functional deficits in drug addiction (117). Therefore, the effects of baclofen on neuroimmune/neurotransmitter systems may participate in a normalization of functional connectivity in patients with AUD.

Indifference to alcohol is a special phenomenon. The case of Olivier Ameisen is very illustrative (3, 14). Ameisen progressively increased the dose of baclofen up to 270 mg/day, and became completely indifferent to alcohol at that dose. At the dose of 260 mg/day, he was not indifferent at all. It is the addition of 10 mg that abruptly and completely changed his attitude toward alcohol. The long experience of baclofen prescription in AUD shows that this abrupt occurrence of a state of indifference at a given dose is common. Patients call it “my threshold.” The threshold of indifference is unique to each patient. Some reach it at moderate doses, some at high or very high doses. The passage from a state of extreme vulnerability to compulsive drinking to a state of indifference is however not always as abrupt as in the case of Olivier Ameisen. Instead, it is often preceded by a period of a slow decrease of craving; but almost all patients who reach a state of indifference say that at a certain dose they felt a complete change in their attitude toward alcohol. For the majority of patients, baclofen treatment is a quest to reach the threshold of indifference. In a recently published guidance for baclofen treatment of AUD, primarily written by expert patients, the quest for the threshold of indifference was clearly described (79). In this guidance article, the authors present what they call the “Ameisen test:” “One of the best ways to confirm that the effective treatment dose has been reached is to ask the patient to go to the shop where s/he used to buy alcohol. If the desire to drink alcohol is ignited by the sight of wine and spirits, the baclofen dose should continue to be increased progressively. If the sight of alcohol has no more effect than looking at nappies or washing powder, the effective dose of baclofen has been reached.” Patients indifferent to alcohol are no longer concerned or stressed by the sight of alcohol. Alcohol has become devoid of meaning. It is well established that craving and the subsequent sequence of compulsive drinking are triggered by feelings of stress that can themselves be triggered by the exposition to alcohol cues (118). The analysis of the mechanisms potentially involved in the dose-dependent, and often abrupt, passage from a state of extreme vulnerability to compulsive drinking to a state of complete indifference is critical in addressing the question of the mechanism of action of baclofen in the treatment of AUD.

From a clinical/phenomenological standpoint, craving and compulsive drinking can be assimilated to a Pavlovian reaction where stress or the activation of an alcohol-related cue or mental imagery is associated with the memory of a reward, and triggers an irrepressible drinking behavioral sequence. The learning of cue-reward associations is a slow process causing long-lasting synaptic plasticity changes in cortico-limbic-striatal circuitry, via multiple gene and protein expression. It is well established that there is a very important relation between stress and alcohol use (34, 35). The biological bases of craving and drinking in response to stress or alcohol cues have been extensively studied in preclinical models of alcohol addiction and in AUD patients themselves. Interestingly, endogenous substances like dopamine and corticotropin-releasing factor and exogenous acute and chronic ethanol act in brain regions that regulate stress and anxiety-related behaviors (119), the most important region being the amygdala. The amygdala is a critical part of the reward network, involved in the way cues associated with rewards gain access to regions attributing incentive salience (120). The way stress and cues associated with rewards are processed in the amygdala may therefore determine subsequent behaviors such as compulsive drinking. Baclofen can interfere with these processes. Baclofen affects memory processes in rodent addiction models, impairing consolidation of memory (121), facilitating extinction learning (122), and interfering with fear extinction (123). Amygdala CREB is known to be involved in the modulation of fear memory (124); and baclofen suppresses stimulant-induced increases in pCREB levels in the amygdala (125). Progressive increase in the reinforcing effects of drug cues is associated with the increases in BDNF and extracellular signal regulated kinase (ERK) activity in the central nucleus of the amygdala (126–128); and GABA-B receptors are involved in the regulation of BDNF release (129), and of the ERK pathway (130). Baclofen also has important neuromodulatory effects in the amygdala, through its inhibitory action on neurotransmitters and complex effects on second-messenger signaling (37). It reduces the strength of excitatory (glutamate) and inhibitory (GABA) transmission in the amygdala by a presynaptic mechanism (131). Furthermore, as mentioned previously, alcohol addiction is associated with impaired GABA clearance and increased GABA tone in the amygdala, associated, in turn, with higher anxiety-like behavior (30). GABA-B receptor stimulation, which inhibits GABA transmission, should therefore be useful in the treatment of alcohol dependence and associated anxiety (Table 1).

Chronic neuromodulatory effects of baclofen in the amygdala may change the processing of stress and cues, and ultimately alter the functional connectivity within the reward network, in such a way that cues associated with rewards lose their meaning. The dose of baclofen needed to achieve this effect could be very variable from one subject to another in relation to each individual's variable strength of the Pavlovian association between the cue and the reward. In other words, the dose of baclofen would be that which is necessary to overcome the strength of a long-lasting associative learning “carved” in the limbic memory. This could be in accordance with studies that show that higher doses of baclofen are correlated with a higher severity of addiction (8, 132). Clinical experience also shows that when the effective dose is reached—the threshold dose producing a state of indifference—the treatment has to be continued for several months at the same dose before it can be reduced. It is proposed that this delay is necessary to completely suppress the Pavlovian association between the cue and the reward. It has been highlighted that, in patients indifferent to alcohol, those who remain completely free of alcohol for many months are those who will be able to stop taking baclofen, while those who continue to drink, even at moderate levels and without any real desire for alcohol but who do so out of habit or on certain social occasions, will have greater difficulty in stopping baclofen (79). Indeed, in terms of connectivity and synaptic strength, it is likely that the continuation of regular drinking reactivates the Pavlovian association between the cue and the reward every time, making it impossible to suppress, and paving the way for relapse if baclofen is stopped.

## The substitution hypothesis

Alcohol and baclofen produce many similar symptoms or behavioral effects in patients. Both can produce unsteady gait, dizziness, feelings of drunkenness, mood alterations, sensory alterations, confusion, impairment in attention and memory, and sleep disorders, among others (16, 88). Both can also reduce anxiety (89, 90). Patients taking baclofen often spontaneously notice these similarities. Abrupt withdrawal from alcohol and high-dose baclofen may also produce similar symptoms, including confusion, hallucinations, delirium, and seizures (16, 91) (Table 1). The main difference between alcohol and baclofen is that alcohol progressively produces a state of dependence, while this is not the case with baclofen (although a few cases have been reported 133—likely because these are exceptional). Besides, chronic alcohol consumption induces tolerance, whereas tolerance with baclofen is equivocal: as previously mentioned, there is no tolerance to the clinical effectiveness of baclofen in AUD, but there is tolerance to most of its adverse effects (e.g., its effects on locomotor activity) (134).

These clinical similarities raise the possibility that baclofen and alcohol act on the same brain systems, and that baclofen could be a substitution treatment for alcohol dependence. In addictology, a substitution substance is a substance that acts on the same receptors as the targeted substance of abuse. The two substances share many similar effects, but the substitution substance is less prone than the substance of abuse to induce dependence, or not prone at all to do so. Substitution treatments appeared in addictology about 50 years ago for the treatment of opiate addiction. Buprenorphine and other substitution substances used in the treatment of opiate addiction indeed act directly as full agonists, or as agonist/antagonists, on opiate receptors. The problem with baclofen and alcohol is that they do not act on the same receptors. Baclofen is a selective GABA-B agonist, and alcohol has no direct action on GABA-B receptors. However, it is very likely that the two substances indirectly act on the same systems, especially the glutamatergic and GABAergic systems. Even though alcohol does not directly act on GABA-B, it increases GABA(B1) and GABA(B2) receptor expression in different parts of the brain, while baclofen partially reverses these effects (87). Besides, it has been shown that stimulation of presynaptic GABA-B receptors decreases the GABAergic effects of alcohol (86), demonstrating that GABA-B activation can moderate the behavioral sensitivity to alcohol. This has led Clapp et al. (135) to propose that treatment with a GABA-B agonist could substitute for the anxiolytic effect of ethanol, leading to its reduced consumption. Similarly, GABA-B stimulation may substitute the GABA transporter GAT-3 deficiency in the brains of alcoholics, leading to a normalized GABA function in the amygdala (30). More generally, chronic alcohol consumption alters many brain substances, receptors, and pathways (136), including several that interact with GABA-B receptors, such as, among others, the PKA and PKC pathways (137), the Akt pathway (138), the mTORC-1 pathway (139), the ERK1/2 pathway (130), BDNF release (129), or CREB (125): in all these systems, GABA-B stimulation could, in some ways, substitute for the effects of alcohol. The definition of substitution, limited to the notion that substitution should strictly involve substances that act on the same receptors, has been questioned. For instance, Chick and Nutt proposed much more broad and unspecific criteria to define substitution (140). It remains that chronic alcohol consumption impacts many brain systems and that GABA-B may interfere with some of these effects with potential clinical advantages, assimilated to substitution or not.

Another approach to the substitution hypothesis has been proposed by Ameisen (92, 141), who hypothesized that there is a deficit of GHB in the brain of AUD patients. He highlighted the similar behavioral effects of alcohol, baclofen and GHB. Baclofen and GHB share several common neurobiological effects, including GABA-B activation; among alcohol, baclofen and GHB, only GHB occurs naturally in the brain and has brain specific receptors. According to Ameisen, a deficit in brain GHB could cause dysphoria, itself promoting alcohol misuse. Baclofen could be able to compensate the deficit in GHB, thereby suppressing dysphoria and possible dependence (Table 1). Furthermore, GHB itself is used as a treatment of AUD, and it is possible that the effectiveness of GHB is related to its ethanol-mimicking action, making it behave as a substitute for alcohol in the brain (142). Although these are only hypotheses, they exemplify the many ways by which a substance could work as a substitution. However, there are no solid theoretical bases supporting the hypothesis that baclofen is a substitution treatment in AUD.

## Conclusion

This review on the mode of action of baclofen from a clinical standpoint and with a biological perspective highlighted three potential modes of action of baclofen; namely on dopamine, functional connectivity, and as a substitution drug. It is tempting to hypothesize that these approaches are complementary, and that they could be synthesized in the proposition that baclofen may suppress the Pavlovian association between cues and rewards through an action in a critical part of the dopaminergic network (the amygdala), thereby normalizing the functional connectivity in the reward network. It is also proposed that this action is made possible by the fact that baclofen and alcohol act on similar brain systems in certain regions of the brain.

## Author contributions

The author confirms being the sole contributor of this work and has approved it for publication.

### Conflict of interest statement

The author declares that the research was conducted in the absence of any commercial or financial relationships that could be construed as a potential conflict of interest.
